# *Stryphnodendron adstringens* and purified tannin on *Pythium insidiosum*: in vitro and in vivo studies

**DOI:** 10.1186/s12941-017-0183-3

**Published:** 2017-02-23

**Authors:** Rodrigo Trolezi, Juliana Maziero Azanha, Natália Rodrigues Paschoal, Jéssica Luana Chechi, Marcelo José Dias Silva, Viciany Eric Fabris, Wagner Vilegas, Ramon Kaneno, Ary Fernandes Junior, Sandra de Moraes Gimenes Bosco

**Affiliations:** 1 Department of Microbiology and Immunology, Institute of Biosciences of Botucatu, UNESP Univ Estadual Paulista, Botucatu, SP 18618-970 Brazil; 2 School of Veterinary Medicine and Animal Science, UNESP Univ Estadual Paulista, Botucatu, SP Brazil; 3 Institute of Chemistry, UNESP Univ Estadual Paulista, Araraquara, SP Brazil; 4 Department of Pathology, Botucatu School of Medicine, UNESP Univ Estadual Paulista, Botucatu, SP Brazil

**Keywords:** *Pythium insidiosum*, Pythiosis, Susceptibility tests, Oomycete

## Abstract

**Background:**

*Pythium insidiosum* is the etiological agent of pythiosis, an emerging life-threatening infectious disease in tropical and subtropical regions. The pathogen is a fungus-like organism resistant to antifungal therapy, for this reason, most cases need extensive surgical debridments as treatment, but depending on the size and anatomical region of the lesion, such approach is unfeasible. We investigate the fungicidal effect and toxicity of crude bark extract of *Stryphnodendron adstringens* and commercially available tannin on *Pythium insidiosum* both in vitro and in vivo.

**Methods:**

Standardized fragments of mycelia of fifteen isolates of *P. insidiosum* were tested with different concentrations of bark extract (10 to 30% v/v) and tannin (0.5, 1.0 and 1.5 mg/mL). For in vivo study, fifteen rabbits were experimentally infected with zoospores of *P. insidiosum* and treated by oral and intralesional applications of bark extract and tannin. Acute toxicity tests with both substances were also performed in rats.

**Results:**

In vitro studies showed fungicidal effect for both substances at different concentrations and the SEM showed alteration on the cell wall surface of the pathogen. All infected rabbits developed a firm nodular mass that reached around 90 mm^2^ ninety days after inoculation, but neither the intralesional inoculation of tannin, nor the oral administration of crude extract and tannin were able to promote remission of the lesions.

**Conclusions:**

Lesions developed by rabbits presented an encapsulated abscess being quite different of naturally acquired pythiosis, which is characterized by ulcerated lesions. Since no toxicity was observed in rats or rabbits inoculated with these products, while in vitro experiments showed direct antifungal effect, therapeutic activity of *S. adstringens* and tannin should be clinically tested as an alternative for healing wounds in naturally acquired pythiosis.

## Background

Pythiosis is caused by the oomycete *Pythium insidiosum* in different animal species mainly in tropical regions. One of the most important differences between oomycetes and true fungi is the absence of ergosterol in the cell membrane of oomycetes that may explain the failure of conventional therapy since most antifungal drugs acts on this cell compound [1].

Lack of an efficient treatment against this pathogen has stimulated the search for new potentially useful compounds including natural products. One of the pioneering studies using natural compounds against *P. insidiosum* was conducted by Zanette et al. [2], who showed that garlic extract is able to inhibit the zoopospores filamentation.

*Stryphnodendron adstringens*, popularly called barbatimão in Brazil, is a common medicinal plant found in Brazilian Cerrado region [3]. This species belongs to the family Fabaceae and it is widely used in traditional medicine for diarrhoeas, gynaecological problems and for wound healing [4]. In addition, antimicrobial, anti-inflammatory, antiulcerogenic and wound cicatrizing properties of bark extract have been reported [5–8]. These effects are attributed to the major compound, tannin, found in the barks and leaves of this tree that shows fungicidal activity against *Candida albicans, Candida* spp., *Trichophyton rubrum* and *Cryptococcus neoformans* [3, 9, 10].

Based on these previous reports, we hypothesized that *S. adstringens* has bioactive compounds that could be able to fight *P. insidiosum*. Therefore, in this study we evaluated the effect of both crude bark extract of *S. adstringens* and commercially available tannin on the in vitro growth of *P. insidiosum*, as well as their in vivo activity in experimentally infected rabbits.

## Methods

### Bark extract of *Stryphnodendron adstringens*

*Stryphnodendron adstringens* barks were collected in Rubião Junior district (Botucatu, SP, Brazil, 22°52′60′′S and 48°28′60′′W), during the morning period. Voucher specimens were deposited at the Herbarium of the Department of Botany, Institute of Biosciences, UNESP, under the number 026633 BOTU. A crude extract was obtained according to Betoni et al. [11]. Briefly, *S. adstringens* barks were dried, ground and extracted with 70% methanol at 4–8 °C and filtered after 48 h. The residual plant material was re-extracted with methanol 70% and filtered after 24 h. The extract was concentrated in a rotary evaporator at 45 °C for elimination of methanol and kept in sterile flask under refrigeration until use. Levels of total tannin in the extract were measured according to Waterhouse [12], as follows: 10 mg of the extract were dissolved in 50 mL of distilled water, and a 2 mL aliquot was mixed with 2 mL of Folin–Ciocalteu reagent and homogenized. Three minutes later, 2 mL of 8% sodium carbonate solution were added to this mixture, stirred and kept for 2 h at room temperature. Then, centrifugated at 2000 rpm to measure total levels of tannin. The analytical calibration curve of commercially available tannin (Sigma-Aldrich) was estimated by the absorbance at 725 ηm of 10, 20, 30, 40, 60 and 80 µL/mL.

### In vitro antimicrobial effect of bark extract of *S. adstringens* and tannin

Fifteen isolates, obtained from clinical cases in human (B-01) and equines (Eq-2 to Eq-15) occurred in São Paulo State, Brazil, were maintained in Sabouraud (SAB) Agar, at 37 °C/7 days. After this period standardized mycelia fragments of 5 mm of diameter were tested with different concentrations of bark extract and purified tannin. Ten to 30% of bark extract were added to 1.0 mL of SAB broth and *P. insidiosum* hyphae standardized fragments of mycelia were added to these solutions. These fragments were incubated under shaking (100 rpm) at 37 °C/24 h. After that, each fragment was placed individually in SAB agar plates and incubated at 37 °C/7 days in order to follow the hyphal growth and determine the minimal fungicidal concentration (MFC). All tests were performed in quintuplicate. Tannin was tested at concentrations of 0.5, 1.0 and 1.5 mg/mL using the same procedure described for bark extract. Control groups consisted of hyphae standardized fragments incubated in SAB broth and placed in SAB agar. Hyphae fragments at MFC, were cut and fixed with 2.5% glutaraldehyde and then routinely processed for analysis by scanning electron microscopy (SEM) aiming to evaluate the hyphal morphology.

### In vivo acute toxicity test

In order to investigate whether bark extract and tannin administration by oral route would be toxic, we employed the test of acute toxicity, according to Loomis & Haynes [13]. Briefly, we administered 5 g/Kg of bark extract or tannin, by gavage, in male Wistar rats weighting 250 g. For this assay 15 rats were divided in 3 groups and treated with bark extract, tannin or saline solution (control). Animals were observed for five days, when they were anesthetized with zolazepan chloride (Zoletil^®^ 50, Virbac) and euthanized by anesthetic overdose. Blood was used to analyse serum levels of liver and kidney biochemical markers (ALT, AST, alkaline phosphatasis and urea) and complete hemogram. Liver and kidney were also excised and fixed in 10% formaldehyde and routinely processed for histopathological analysis. Rats were kept in cages (5 animals/cage) in the animal facility of the Department of Microbiology and Immunology, Institute of Biosciences, UNESP, and all procedures were approved by the local Ethics Committee on Animal Use (protocol number 536).

### In vivo experimental pythiosis

Fifteen New Zealand rabbits were inoculated with 2 mL of induction medium (as described according to Mendoza and Prendas [14] containing around 20.000 zoospores of *P. insidiosum*), in the occipital region, as described by Pereira et al. [15]. Thirty days after inoculation, the animals were separated into five different groups with 3 animals each for treatment as follows: *S. adst.* = bark extract by oral route (90 mg/day); TAN = intralesional tannin (30 mg/mL each 48 h); TAN-VO = tannin by oral route (60 mg/day); TAN-PRED = intramuscular injection of methylprednisolone (1 mg/Kg, once a week) and intralesional tannin (30 mg/mL, associated with 1% DMSO, each 48 h), and control group (no treatment). Association with DMSO aimed to enhance the tannin diffusion while methylprednisolone was administrated at anti-inflammatory dose. The treatments were followed by macroscopic visual inspection and palpation, and histopathology analysis of HE and Gomori-Grocott stained slides. Transverse and longitudinal lengths were also measured with a caliper to calculate the wound area (mm^2^). Infected rabbits were also analyzed on hepatic and kidney functions through the quantification of biochemical parameters (AST, ALT, alkaline phosphatasis and urea), as well as complete hemogram. Stomach and duodenum of animals treated by oral route were histopatologically analyzed. Rabbits were kept in individual cages at the animal facility of the Departments of Microbiology and Immunology, Institute of Biosciences, UNESP, and all procedures were approved by the local Ethics Committee on Animal Use, UNESP (protocol number 370).

## Results

**Fig. 1:**
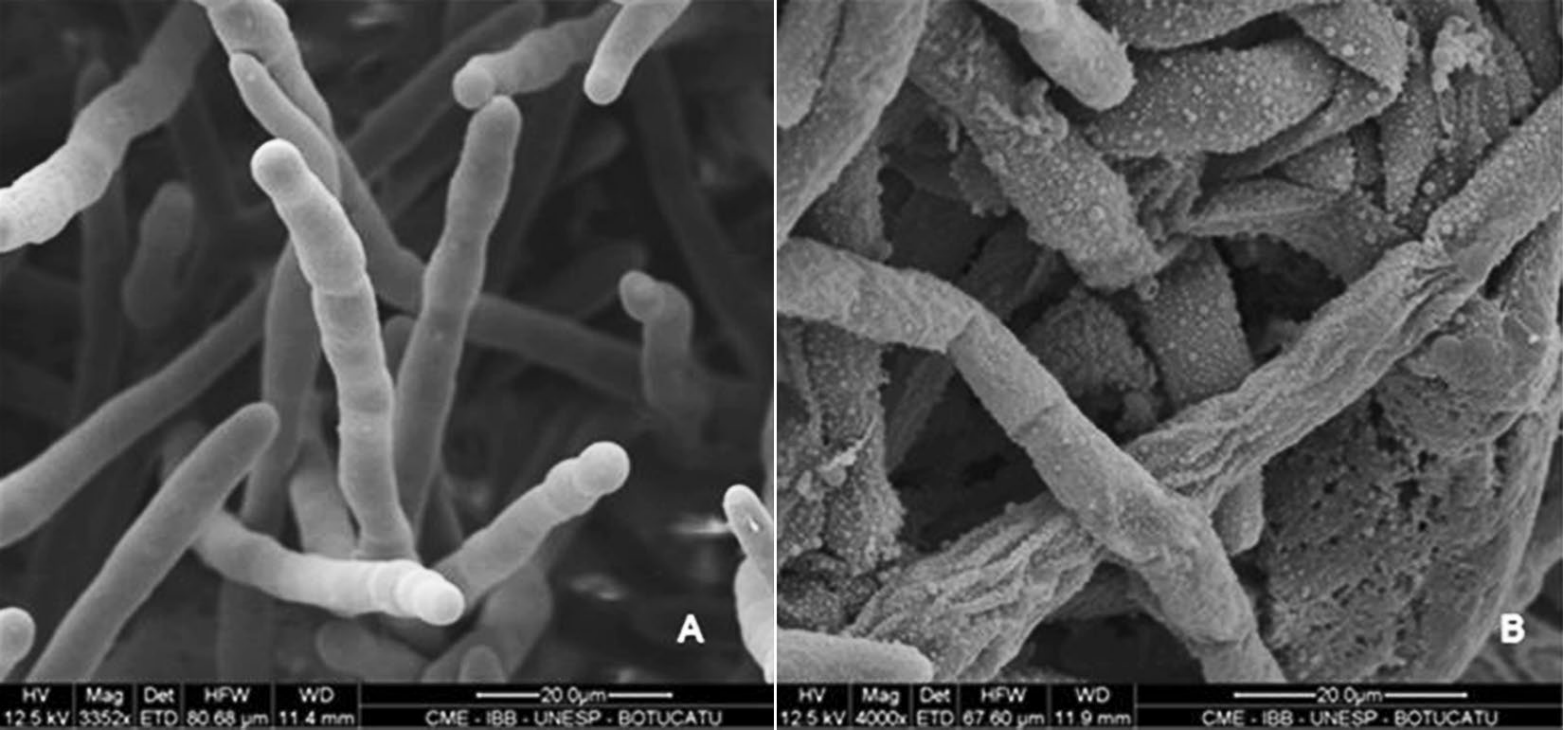
Scanning electron microscopy of *Pythium insidiosum* from control group (**A**) and treated with MFC of bark extract of *S. adstringens* (**B**). Observe the cylindrical morphology of hyphae and smooth surface of cell wall from control group, while the hyphae treated with bark extract show rough surface of cell wall, high amount of granular material and release of anamorphic material

Crude bark extract showed 46.14% of total tannin concentration, which amount was considered for the serial dilution to determine the MFC (Table 1). Three out of 15 *P. insidiosum* isolates (20%) required more than 1.5 mg/mL; ten (66.7%) showed MFC between 1 and 1.5 mg/mL, while only two isolates (13.3%) required less than 1.0 mg/mL of bark extract to be inhibited. In relation of commercially available tannin, six isolates were inhibited at 0.5 mg/mL (40%), three isolates were inhibited at 1.0 mg/mL (20%) and six at 1.5 mg/mL (40%). Figure 1 shows the morphological features of hyphae of control group (Fig. 1A) with cylindrical body and smooth surface. Changes induced by bark extracts at MFC are illustrated at Fig. 1B, showing rough surface, release of anamorphic content and numerous granular material on the cell wall surface.

**Table 1:** Isolates of *Pythium insidiosum* obtained from clinical cases of pythiosis in human (B-01) and horses (Eq-2 to 15), evaluated for minimal fungicidal concentration (MFC) of bark extract (in percentage of v/v, mg/mL of dry weight of extract and mg/mL of total tannin quantification) and purified tannin (mg/mL)

*Pythium insidiosum* isolates	MFC of bark extract			MFC purified tannin (mg/mL)
	% (v/v)	mg/mL of extract, according to dry weight	Total tannins (mg/mL)	
B-01	14	2.80	1.29	1.00
Eq-2	12	2.40	1.10	1.50
Eq-3	20	4.00	1.84	1.50
Eq-4	16	3.20	1.48	1.50
Eq-5	26	5.20	2.40	0.50
Eq-6	17	3.40	1.57	1.00
Eq-7	16	3.20	1.48	1.50
Eq-8	11	2.20	1.01	1.50
Eq-9	16	3.20	1.48	1.00
Eq-10	16	3.20	1.48	1.50
Eq-11	11	2.20	1.01	0.50
Eq-12	10	2.00	0.92	0.50
Eq-13	12	2.40	1.10	0.50
Eq-14	11	2.20	1.01	0.50
Eq-15	10	2.00	0.92	0.50

Toxicity tests made in rats showed that *S. adstringens* extract, as well as tannin, presented no acute toxicity, since no changes were observed in biochemical parameters of liver and kidney functions or animal behavior. Erythrocytes and leukocytes countings were also similar to the control animals. Administration of bark extract or tannin solution in rabbits seems to be also free of toxic effects on liver, kidney and blood system (Table 2).

**Table 2:** Erythrocytes, white blood cells and plasma biochemical values of rats and rabbits from control and treatment groups (mean ± SD)

Animals	Treatments	RBC X10^6^ cells/µL	WBC X10^6^ cells/µL	ALT U/I	AST U/I	ALF U/I	Urea mg/dL	Creatinine mg/dL
Rats	Control	7.34 ± 0.47	3.1 ± 0.27	20.38 ± 1.41	125.00 ± 0.26	71.15 ± 0.31	71.30 ± 0.34	9.37 ± 0.57
	Bark extract	7.38 ± 0.47	2.96 ± 0.11	20.90 ± 1.61	123.8 ± 0.98	70.71 ± 0.58	71.12 ± 0.76	9.06 ± 0.29
	Tannin	7.35 ± 0.42	2.98 ± 0.16	20.04 ± 1.12	123.6 ± 0.89	70.62 ± 0.54	71.14 ± 0.86	9.30 ± 0.42
Rabbits	Control	5.63 ± 0.80	6.33 ± 1.06	71.39 ± 1.61	53.58 ± 0.76	25.27 ± 0.26	23.47 ± 1.20	1.22 ± 0.23
	*S. adst*	6.13 ± 1.06	6.46 ± 1.35	72.27 ± 2.07	53.53 ± 1.35	24.54 ± 0.61	23.17 ± 0.30	0.87 ± 0.14
	TAN	6.20 ± 1.11	6.70 ± 0.80	71.94 ± 1.99	53.14 ± 1.53	24.16 ± 1.09	23.13 ± 0.15	1.08 ± 0.12
	TAN-VO	6.53 ± 1.00	6.86 ± 1.30	71.66 ± 1.43	53.95 ± 1.39	23.74 ± 1.53	22.96 ± 0.42	0.96 ± 0.09
	TAN-PRED	6.23 ± 0.92	6.56 ± 0.85	72.16 ± 0.60	52.16 ± 0.60	23.46 ± 1.43	22.87 ± 1.07	0.97 ± 0.13

*RBC* red blood cells, *WBC* white blood cells, *ALT* alanine aminotransferase, *AST* aspartate aminotransferase, *ALF* alkaline phosphatasis

**Fig. 2:**
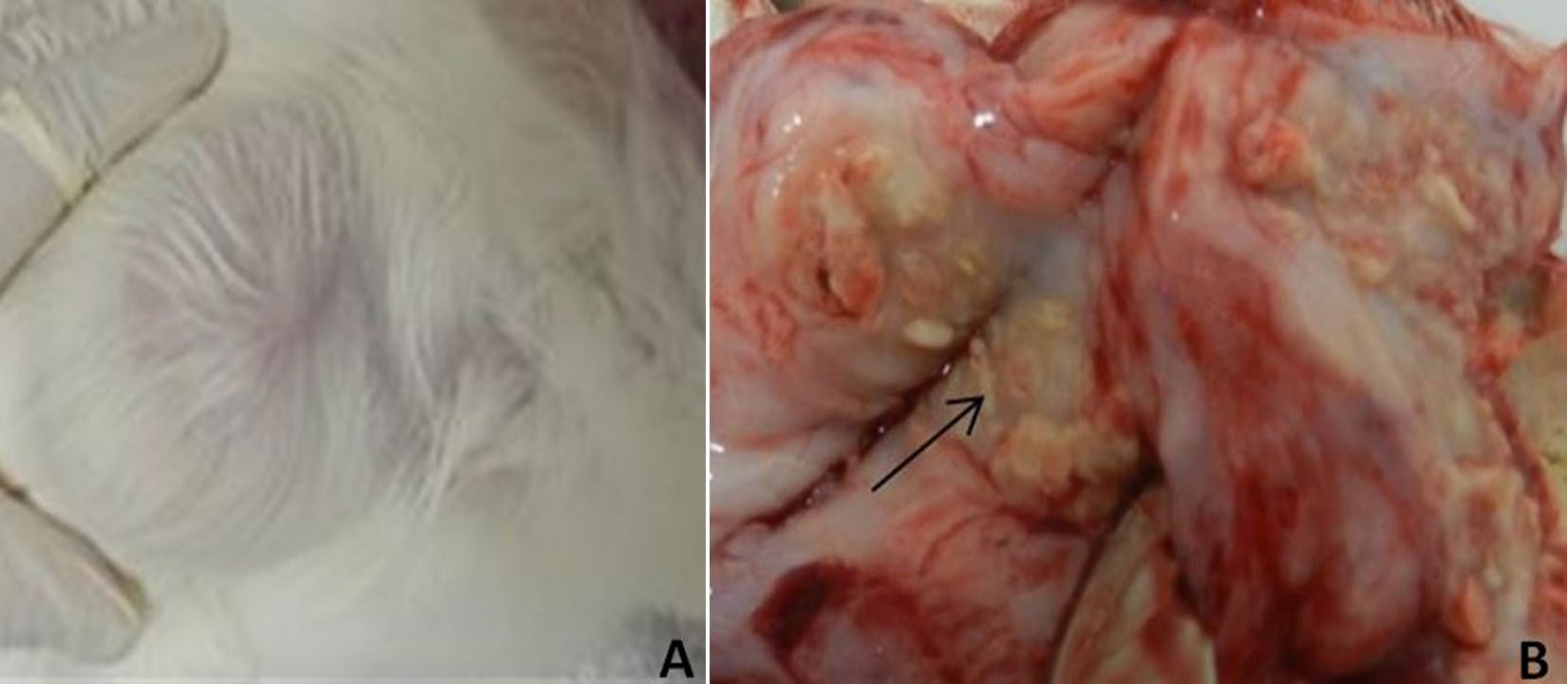
Macroscopic aspect of encapsulated abscess in rabbit experimental model. **A** External view, **B** internal view of the abscess exhibiting caseous yellowish–green material in the center (*arrow*) surrounded by fibrous connective tissue

**Fig. 3:**
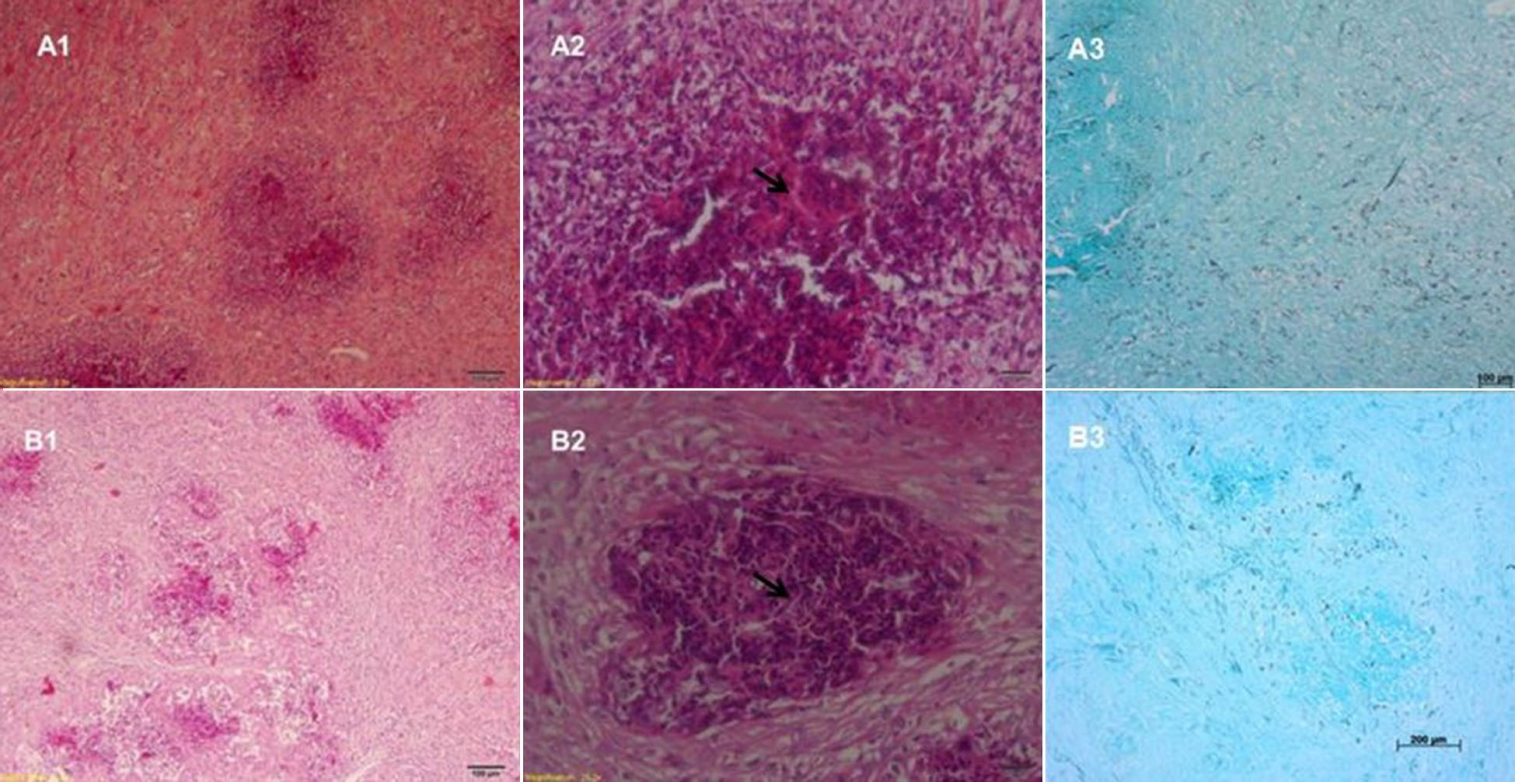
Histological section of one animal from control group (**A**) and *S. adst.* group (**B**). *(1)* general view of both treatments (Hematoxilin–Eosin, 100×), *(2)* presence of hyphae and defense cells, with predominance of neutrophils and eosinophils (Hematoxilin–Eosin, 200×), *(3)* general view of granuloma showing high amounts of hyphae (Gomori-Grocott, 100×)

All rabbits developed lesions at the inoculation site. Overall, the lesions appeared 20–30 days after inoculation and were characterized by encapsulated abscess with a firm consistency. Table 3 shows the mean size of the lesions before and at the end of treatment, indicating that there were no differences between the control group and any therapeutic approach we used. Macroscopic evaluation showed large granulomatous lesion containing caseous necrotic material with yellowish-green pus (Fig. 2). Microscopically we observed the typical eosinophilic granuloma with abundance of fibrous connective tissue and numerous hyphal elements evidenced by silver staining (Fig. 3).

**Table 3:** Transverse and longitudinal lengths of the rabbit pythiosis lesions before and after 60 days of the different treatment protocols

Groups	Measurement before treatment (mm)	Measurement after treatment (mm)
*S. adst.*	16 × 20	85 × 88
TAN	25 × 26	90 × 91
TAN-VO	17 × 17	87 × 89
TAN-PRED	18 × 22	85 × 89
Control	21 × 17	89 × 89

## Discussion

Antimicrobial activity of *S. adstringens* has been shown on *Candida* spp., *Cryptococcus neoformans*, some dermatophytes, viruses, bacteria and protozoa [3, 8–10, 16, 17].

It antimicrobial activity combined with the healing action leaded us to choose this medicinal plant to investigate its therapeutic potential against *P. insidiosum* and pythiosis.

We first evaluated the in vitro effect of bark extract and tannin on this pathogen and observed that both were effective in inhibiting the mycelial growth. The tannin content in the bark extract was 46.14% and it was enough to inhibit the in vitro growth of pathogen. It has been reported that tannin may promote the precipitation of protein and/or polysaccharides, leading to formation of tannin-protein and/or tannin-polysaccharides complexes, justifying its antimicrobial effect [18]. Morphological analysis by SEM revealed several granular precipitations on the cell wall surface that may be the polysaccharide complexes. Cell wall of this pathogen is composed mainly by β-glucan and cellulose (polysaccharides), which may be one of the targets for tannins.

There is no standardized technique for in vitro studies of *P. insidiosum* and the few studies reporting tests of susceptibility of this pathogen were carried out with the zoospores, a form found in the environment [15, 19, 20]. Studies on the hyphal form of the pathogen are scarce and should be more stimulated, since they are simple and reproducible [21]. Regarding the susceptibility of this pathogen to natural compounds, Zanette et al. [2] observed that garlic extracts inhibited the formation of germinative tube in the zoospores of seventeen isolates of *P. insdiosum*. Krajaejun et al. [22] observed that the volatile organic compound obtained from the endophytic fungus *Musdocor crispans* was able to inhibit the mycelia growth of thirty isolates of *P. insidiosum*. Sriphana et al. [23] evaluated the roots of a traditional medicinal plant in Thailand, *Alyxia schlechteri* and found three new lignan esters, called alyterinates A–C [1–3], which had an inhibitory effect on hyphal growth, while terbinafine and itraconazole failed to show antifungal effect. Additional study carried out by these authors also found that the alkaloid clauraila E, obtained from *Clauseana harmandiana* roots, another traditional medicinal plant in Thailand, was able to inhibit mycelia growth [24]. More recently, Suthiwong et al. [25] found four new compounds from the fruits of *Micromelum falcatum* able to inhibit the mycelia growth of *P. insidiosum*. Fonseca et al. [26] evaluated the effect of essential oils of Lamiaceae Family and observed that *Origanum vulgare* showed the best performance in inhibiting the germination of zoospores of *P. insidiosum*. Therefore, it seems that new compounds with potential therapeutic activity against pythiosis may come from nature. In this sense, Fonseca et al. [27] showed that topical cream with essential oils of *O. vulgare* and *Mentha piperita* enhances the effect of immunotherapy to reduce the size of pythiosis lesions.

Few studies on pythiosis were conducted both in vitro and in vivo. Pereira et al. [15] evaluated the effect in vitro and in vivo of caspofungin, who administered it by intravenous rout and observed only fungistatic effect of this antifungal agent. Likewise, Loreto et al. [28] evaluated the effect of diphenyl diselenide in vitro and in vivo administrated by oral route, and also observed fungistatic effect of this compound and no toxicity.

Our in vivo toxicological evaluation of bark extract and purified tannin in Wistar rats showed no signs of toxicity in the histologic sections of liver and kidney or biochemical parameters of hepatic and renal functions. No hepatic or renal dysfunctions were observed in rabbits either. These results are a little bit different of those reported by Costa et al. [29] who evaluated the acute toxicity of different concentrations of bark extract in Swiss mice and rats, and observed more toxicological effects in mice than in rats.

Despite of being very effective in vitro and showed no toxicity in vivo, the application of bark extract or tannin in the experimental model of pythiosis were not as satisfactory as expected. In all rabbits the main macroscopic aspect of lesion was of encapsulated abscess, with very rigid fibrous connective tissue. At necropsy it was observed caseous necrotic material with focus of yellowish–green pus, in agreement with Miller and Campbel [30], pioneers of experimental studies on pythiosis. We believe that this encapsulated abscess is the most important reasons for low perfusion of tannin administrated intralesionally, which provides a physical barrier to medications, even when associated with dimethyl sulphoxide (DMSO, a potent organic solvent used to facilitate the perfusion) and systemic administration of methylpredinisolone (used in anti-inflammatory dose). Actually, this barrier had difficulted the needle insertion for in situ inoculation of the solutions and should also avoid the perfusion of orally administrated substances.

Therefore, an important difficulty for evaluating new approaches for treatment in vivo especially topical ones, is that lesions observed in experimental disease in rabbits is very different of naturally acquired disease. Horses and dogs, for example, develop ulcerated subcutaneous lesions, while in rabbits the lesions are well encapsulated in a fibrous abscess. Then, it is possible that the effectivity of *S. adstringens* extracts against *P. insidiosum* lesions would be observed in a more adequate model.

## Conclusion

Our results allow us to conclude that both bark extract and purified tannin have direct fungicidal effect on hyphal growth of different isolates of *P. insidiosum*. Despite we were not able to demonstrate a therapeutic effect in our experimental model, the abcense of toxicity in addition to the lack of efficient conventional medications for pythiosis, it would be ethically acceptable to test these compounds topically as alternative treatment of naturally infected horses and dogs.

## Abbreviations

SAB: Sabouraud (agar and broth)
MFC: minimal fungicidal concentration
AST: aspartate aminotransferase
ALT: alanine aminotransferase
HE: Hematoxylin–Eosin stain

## References

[1] Gaastra W, Lipman LJA, De Cock AWAM, Exel TK, Pegge RBG, Scheurwater J, Vilela R, Mendoza L (2010). *Pythium insidiosum*: an overview. Vet Microbiol.

[2] Zanette RA, Bittencourt PER, Weiblen C, Pilotto MB, Pigatto AS, Ceolin RB, Moretto MB, Alves SH, Santurio JM (2011). *In vitro* susceptibility of *Pythium insidiosum* to garlic extract. J Afr Microbiol Res..

[3] Melo Silva F, de Paula JE, Espindola LS (2009). Evaluation of the antifungal potential of Brazilian Cerrado medicinal plants. Mycoses.

[4] Nunes GP, Silva MF, Resende UM, Siqueira JM (2003). Plantas medicinais comercializadas por raizeiros no Centro de Campo Grande, Mato Grosso do Sul. Rev Bras Farmacogn..

[5] Audi EA, Toledo DP, Peres PG, Kimura E, Pereira WK, de Mello JC, Nakamura C, Alves-do-Prado W, Cuman RK, Bersani-Amado CA (1999). Gastric antiulcerogenic effects of *Stryphnodendron adstringens* in rats. Phytother Res..

[6] Hernandes L, Pereira LMS, Palazzo F, Mello JCP (2010). Wound-healing evaluation of ointment from *Stryphnodendron adstringens* (barbatimão) in rat skin. Braz J Pharmac Sci..

[7] Pereira EM, Gomes RT, Freire NR, Aguiar EG, Brandão MD, Santos VR (2011). *In vitro* antimicrobial activity of Brazilian medicinal plant extracts against pathogenic microorganisms of interest to dentistry. Planta Med.

[8] Fernandes A, Albano M, Alves FCB, Andrade BFMT, Barbosa LN, Silva GS, Di Stasi LC (2014). Medicinal plants from the Brazilian Savanna with antibacterial properties. Euro J Med Plants..

[9] Ishida K, Melo JCP, Cortez DAG, Dias Filho BP, Ueda-Nakamura T, Nakamura CV (2006). Influence of tannins from *Stryphnodendron adstringens* on growth and virulence factors of *Candida albicans*. J Antimicrob Chemother.

[10] Ishida K, Rozental S, de Mello JC, Nakamura CV (2009). Activity of tannins from *Stryphnodendron adstringens* on *Cryptococcus neoformans*: effects on growth, capsule size and pigmentation. Ann Clin Microbiol Antimicrob..

[11] Betoni JEC, Mantovani RP, Barbosa LN, Di Stasi LC, Fernandes Junior A (2006). Synergism between plant extract and antimicrobial drugs used on *Staphylococcus aureus* diseases. Mem Inst Oswaldo Cruz.

[12] Waterhouse AL, Wrolstad RE (2002). Polyphenolics: Determination of total phenolics. Current protocols in food analytical chemistry.

[13] Loomis TA, Hayes AW (1996). Loomis’s essentials of toxicology.

[14] Mendoza L, Prendas J (1988). A method to obtain rapid zoosporogenesis of *Pythium insidiosum*. Mycopathologia.

[15] Pereira DIB, Santurio JM, Alves SH, Argenta JS, Pötter L, Spanamberg A, Ferreiro L (2007). Caspofungin in vitro and in vivo activity against Brazilian *Pythium insidiosum* strains isolated from animals. J Antimicrob Chemoth..

[16] Holetz FB, Ueda-Nakamura T, Dias Filho BP, Mello JCP, Morgado-Díaz JA, Toledo CEM, Nakamura CV (2005). Biological effects of extracts obtained from *Stryphnodendron* *adstringens* on *Herpetomonas samuelpessoai*. Mem Inst Oswaldo Cruz.

[17] Felipe AM, Rincão VP, Benati FJ, Linhares RE, Galina KJ, de Toledo CE, Lopes GC, de Mello JC, Nozawa C (2006). Antiviral effect of *Guazuma ulmifolia* and *Stryphnodendron* *adstringens* on poliovirus and bovine herpesvirus. Biol Pharm Bull.

[18] Santos SC, Costa WF, Ribeiro JP, Guimarães DO, Ferri PH, Ferreira HD, Seraphin JC (2002). Tannin composition of barbatimão species. Fitoterapia.

[19] Sekhon AS, Padhye AA, Garg AK. *In vitro* sensitivity of *Penicillium marneffei* and *Pythium insidiosum* to various antifungal agents. Eur J Epidemiol. 1992. http://www.jstor.org/stable/3520676. 10.1007/BF00158578 1397206

[20] Argenta JS, Santurio JM, Alves SH, Pereira DI, Cavalheiro AS, Spanamberg A, Ferreiro L (2008). *In vitro* activities of voriconazole, itraconazole and terbinafine alone or in combination against *Pythium insidiosum* isolates from Brazil. Antimicrob Agents Chemother.

[21] Brown TA, Grooters AM, Hosgood GL (2008). *In vitro* susceptibility of *Pythium* *insidiosum* and *Lagenidium* sp. to itraconazole, posaconazole, voriconazole, terbinafine, caspofungin, and mefenoxam. Am J Vet Res.

[22] Krajaejun T, Lowhnoo T, Yingyong W, Rujirawat T, Fucharoen S, Strobel GA (2012). *In vitro* antimicrobial activity of volatile organic compounds from *Muscodor crispans* against the pathogenic oomycete *Pythium insidiosum*. SE Asian J Trop Med..

[23] Sriphana U, Thongsri Y, Ardwichai P, Poopasit K, Prariyachatigul C, Simasathiansophon S, Yenjai C (2013). New lignan esters from *Alyxia schlechteri* and antifungal activity against *Pythium insidiosum*. Fitoterapia.

[24] Sriphana U, Thongsri Y, Prariyachatigul C, Pakawatchai C, Yenjai C (2013). Clauraila E from the roots of *Clausena harmandiana* and antifungal activity against *Pythium insidiosum*. Arch Pharm Res..

[25] Suthiwong J, Sriphana U, Thongsri Y, Promsuwan P, Prariychatigul C, Yenjai C (2014). Coumarinoid from the fruits of *Micromelum facatum*. Fitoterapia.

[26] Fonseca AOS, Pereira DIB, Botton SA, Pötter L, Sallis ESV, Júnior SFV, Filho FSM, Zambrano CG, Maroneze BP, Valente JSS, Baptista CT, Braga CQ, Dal Ben V, Meireles MCA (2015). Treatment of experimental pythiosis with essential oils of *Origanum vulgare* and *Mentha piperita* singly, in association and in combination with immunotherapy. Vet Microbiol.

[27] Fonseca AOS, Pereira DIB, Jacob RG, Maia Filho FS, Oliveira DH, Maroneze BP, Valente JSS, Osório LG, Botton SA, Meireles MCA (2015). *In vitro* susceptibility of Brazilian *Pythium insidiosum* isolates to essential oils of some Lamiaceae Family species. Mycopathologia.

[28] Loreto ES, Alves SH, Santurio JM, Nogueira CW, Zeni G (2012). Diphenyl diselenide in vitro and in vivo activity against the oomycete *Pythium insidiosum*. Vet Microbiol.

[29] Costa MA, Mello JCP, Kaneshima EN, Ueda-Nakamura T, Dias Filho BP, Audi EA, Nakamura CV (2013). Acute and chronic toxicity of an aqueous fraction of the stem bark of *Stryphnodendron adstringens* (barbatimão) in rodents. Evid Based Complement Alternat Med..

[30] Miller RI, Campbell SF (1983). Experimental pythiosis in rabbits. Sabouraudia.

